# *Terminalia paniculata* bark extract attenuates non-alcoholic fatty liver via down regulation of fatty acid synthase in high fat diet-fed obese rats

**DOI:** 10.1186/1476-511X-13-58

**Published:** 2014-03-29

**Authors:** Mopuri Ramgopal, Banavathy S Kruthika, Damineni Surekha, Balaji Meriga

**Affiliations:** 1 Department of Biochemistry, Sri Venkateswara University, Tirupati 517502, AP, India; 2 Department of Molecular Reproduction, Development and Genetics, Indian Institute of Science, Bangalore, India

**Keywords:** High fat diet, Liver lipid profile, Superoxide dismutase, Catalase, Malondialdehyde, *FAS*, *AMPK-1α*, Histopathology

## Abstract

**Background:**

This study was performed to understand the possible therapeutic activity of *Terminalia paniculata* ethanolic extract (TPEE) on non alcoholic fatty liver in rats fed with high fat diet.

**Methods:**

Thirty six SD rats were divided into 6 groups (*n* = 6): Normal control (NC), high fat diet (HFD), remaining four groups were fed on HFD along with different doses of TPEE (100,150 and 200 mg/kg b.wt) or orlistat, for ten weeks. Liver tissue was homogenized and analyzed for lipid profiles, activities of superoxide dismutase (SOD), catalase (CAT) and malondialdehyde (MDA) content. Further, the expression levels of *FAS* and *AMPK-1α* were also studied in addition to histopathology examination of liver tissue in all the groups.

**Results:**

HFD significantly increased hepatic liver total cholesterol (TC), triglycerides (TG), free fatty acids (FFA) and MDA but decreased the activities of SOD and CAT which were subsequently reversed by supplementation with TPEE in a dose-dependent manner. In addition, TPEE administration significantly down regulated hepatic mRNA expression of *FAS* but up regulated *AMPK-1α* compared to HFD alone fed group. Furthermore, western blot analysis of *FAS* has clearly demonstrated decreased expression of *FAS* in HFD + TPEE (200 mg/kg b.wt) treated group when compared to HFD group at protein level.

**Conclusions:**

Our biochemical studies on hepatic lipid profiles and antioxidant enzyme activities supported by histological and expression studies suggest a potential therapeutic role for TPEE in regulating obesity through *FAS*.

## Background

Obesity is a well known risk factor for a variety of life style disorders, such as type 2 diabetes, hyperlipidemia, atherosclerosis, cardiovascular diseases and certain cancers [1,2]. It is characterized by accumulation of excess fat in the body and dysregulation of lipid metabolism [3]. The role of fatty acid synthase is implicated in the regulation of fatty acid synthesis and net accumulation of lipid in liver and adipose tissue [4]. Previous studies showed that high fat diets result in non- alcoholic hepatic steatosis (NAHS). Nearly 70-90% of the non alcoholic fatty liver problems were observed in obese and diabetic patients. The NAHS is characterized by accumulation of excessive triacylglycerol (TAG) within hepatocytes and the condition can progress into more serious liver diseases, such as liver fibrosis, cirrhosis, non alcoholic steatohepatites [4-6]. A few pharmaceutical synthetic drugs, fibrates and statins are available to treat obesity. However due to adverse side effects they are not preferred for long time usage [7]. Therefore, attempts are being made to develop natural product-based safe and effective drugs to treat obesity [8,9].

*Terminalia paniculata* Roth. (Combretaceae) is a tropical tree with a large natural distribution in western and southern parts of India. Traditionally, flower juice and bark of *T. paniculata* have been used to treat cholera, inflammation of parotid glands and menstrual disorders [10], cough, bronchitis, cardiac debility, hepatitis and diabetes and it has spermicidal activity too [11-13]. Phytochemicals such as Ellagic acid, Dimethylellagic acid, Pentamethyl flavellagic acid, Trimethyl flavellagic acid and β-Sitosterol have been isolated from the heartwood of *T. paniculata*. The bark was reported to contain 14% tannins which have a pyrogallol nucleus along with gallic acid [14]. The anti oxidant activity of this plant was evaluated with DPPH (2, 2 Diphenyl-1-picrylhydrazyl) by Shalu et al. [15]. Till now there are only few reports about the therapeutic activity of *Terminalia paniculata* and its phytochemicals, but there are hardly any reports in understanding the anti-obesity activity of this plant. Hence, our present work was aimed to investigate the protective effect of TPEE in non alcoholic fatty liver development and to understand the role of genes such as *FAS* and *AMPK-1α* in HFD-fed obese rats.

## Results

### Measurement of body and organ weights

After the end of the experimental period, rats were weighed and body weights noted. HFD-fed rats showed substantial raise in body weight gain, compared to normal control group. However, HFD + TPEE (100, 150 and 200 mg/kg b.wt) administered rats showed significant decrease in weight gain compared to HFD control group (Table 1). Table 1 compares and explains body and organ weights of different groups. The liver and kidney weights of HFD-fed rats increased considerably which were significantly (*p* < 0.05) and dose dependently reduced by TPEE treatment. Whereas spleen and testis of TPEE treated groups did not show any significant variation compared to HFD control.

**Table 1:** Effect of TPEE on body and organ weights of control and HFD-fed rats

**Groups**	**Liver**	**Kidney**	**Spleen**	**Testis**	**Body weight**
NC	8.169 ± 1.2	1.693 ± 0.5	0.593 ± 0.2	2.443 ± 0.6	235 ± 3.02
HFD	11.73 ± 1.3^#^	2.884 ± 0.4^#^	0.664 ± 0.1	2.83 ± 0.4	460 ± 1.26^#^
ORL	7.462 ± 1.1^*^	1.80 ± 0.5^*^	0.5 ± 0.3	2.698 ± 0.8	340 ± 2.41^*^
TPEE100	9.919 ± 0.5	2.97 ± 0.3	0.502 ± 0.2	2.803 ± 0.5	425 ± 5.01
TPEE150	8.69 ± 0.5^*^	2.0 ± 0.3^*^	0.50 ± 0.2	2.803 ± 0.5	380 ± 1.49^*^
TPEE200	7.01 ± 0.5^*^	1.62 ± 0.5^*^	0.50 ± 1.5	2.71 ± 0.9	350 ± 1.10^*^

The data are given as mean ± S.D (*n* = 6). ^*^(*P* < 0.05). Values are tested by ANOVA with Duncan’s multiple range tests. ^#^*P* < 0.05 significantly different from normal control, ^*^P < 0.05 significantly different from HFD control NC: Normal control group, HFD: high-fat diet group, ORL: Orlistat, TPEE: *T.Paniculata* ethanolic extract (100,150 and 200 mg/kg b.wt/day).

### Hepatic lipid profiles

To determine the effect of TPEE on hepatic lipid profiles, we estimated total cholesterol (TC), triglycerides (TG) and free fatty acids (FFA) in all groups of rats. Table 2 compares and explains lipid profiles of different groups. TPEE + HFD (100, 150 and 200 mg/kg b.wt) administered rats showed reduced TC (12.5%, 26.5% and 39%), TG (15%, 26% and 42%) and FFA (16%, 35% and 56%) levels when compared to HFD fed control group (Table 2). The above results demonstrated that TPEE at a dose of 200 mg/kg b.wt showed similar activity as that of HFD + orlistat treated group.

**Table 2:** Effect of TPEE on liver lipid levels of control and HFD-fed rats

**Groups**	**Total cholesterol**	**Triglycerides**	**Free fatty acids**
NC	2.32 ± 0.25	78.83 ± 6.85	5.8 ± 1.15
HFD	4.60 ± 0.25^#^	171.8 ± 9.2^#^	12.6 ± 2.4^#^
ORL	2.38 ± 0.21^*^	90 ± 11.0^*^	4.42 ± 1.17^*^
TPEE100	4.10 ± 0.24	151 ± 10.7	10.5 ± 1.70
TPEE150	3.67 ± 0.27^*^	137.6 ± 6.2^*^	8.5 ± 1.67^*^
TPEE200	2.87 ± 0.15^*^	99 ± 10.8^*^	5.5 ± 2.12^*^
F Value	98.830	95.489	19.721
Significance	0.000	0.000	0.000

The data are expressed as the mean ± S.D (*n* = 6). Values are tested by ANOVA with Duncan’s multiple range tests. ^#^*P* < 0.05 significantly different from normal control, ^*^P < 0.05 significantly different from HFD control. NC: Normal control group, HFD: high-fat diet group, ORL: Orlistat, TPEE: *T. Paniculata* ethanolic extract (100, 150 and 200 mg/kg/day).

### Activity of liver antioxidant enzymes

**Figure 1:**
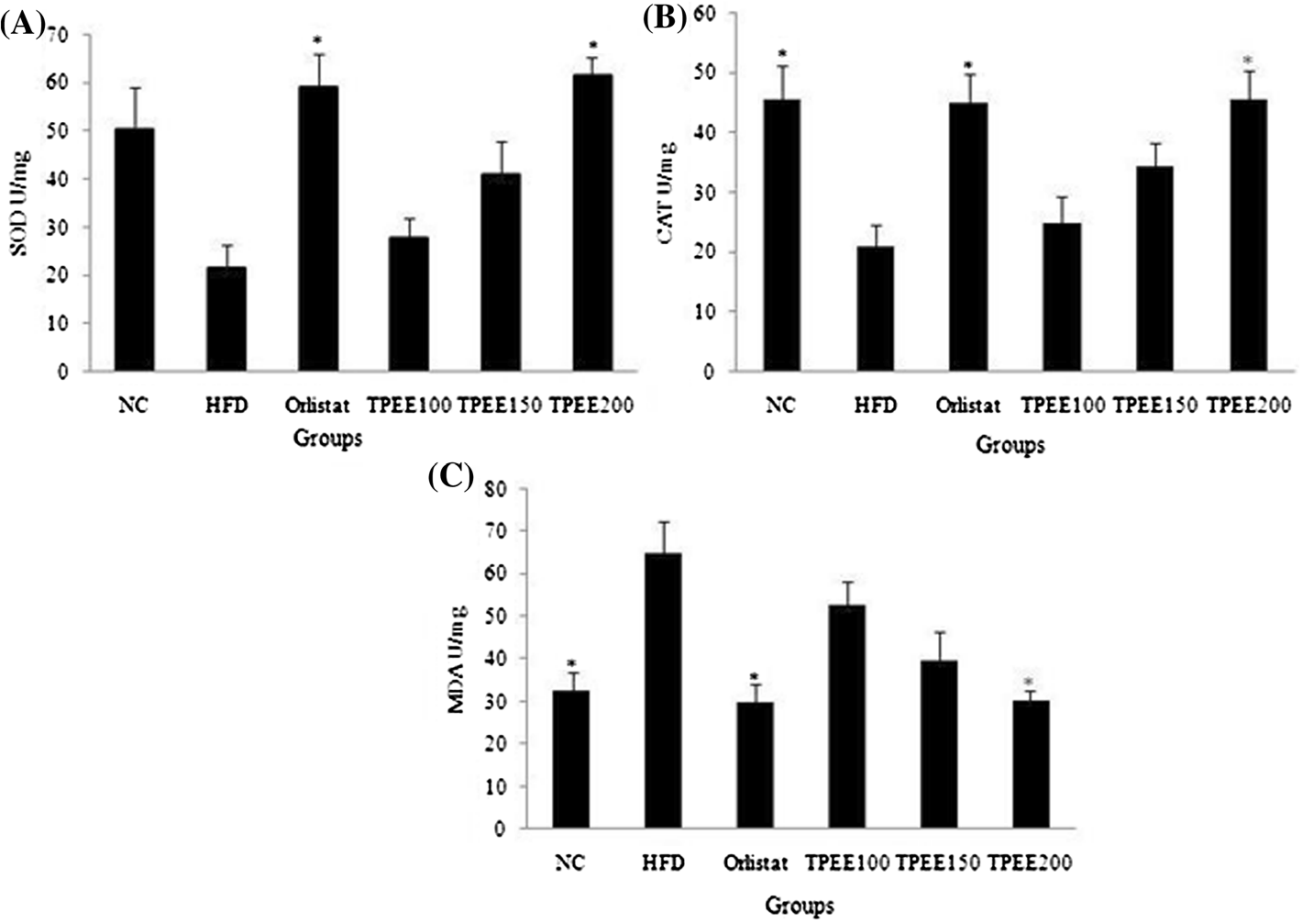
**Effect of TPEE on (A) SOD activity (B) CAT activity and (C) MDA content in liver.** The data are expressed as the mean ± S.D (*n* = 6). Values were tested by ANOVA with Duncan’s multiple range test. ^*^P < 0.05 significantly different from HFD control. NC: Normal control group, HFD: high-fat diet group, TPEE: *T. paniculata* ethanolic extract (100,150 and 200 mg/kg b.wt/day).

To determine the in vivo antioxidant activity, the levels of superoxide dismutase (SOD), catalase (CAT) and malondialdehyde (MDA) content were estimated from the liver tissue. There was a substantial decrease in the activity of SOD and CAT but raise in MDA content in HFD-fed groups. However, TPEE (100, 150 and 200 mg/kg b.wt) administration has significantly reversed these alterations in SOD, CAT and MDA levels. At a dose of 200 mg/kg b.wt, TPEE increased the activity of SOD and CAT by 62% and 53% respectively but decreased MDA content by 49% as shown in Figure 1.

### Effect of TPEE on expression of *FAS* and *AMPK-1α* by RT-PCR

**Figure 2:**
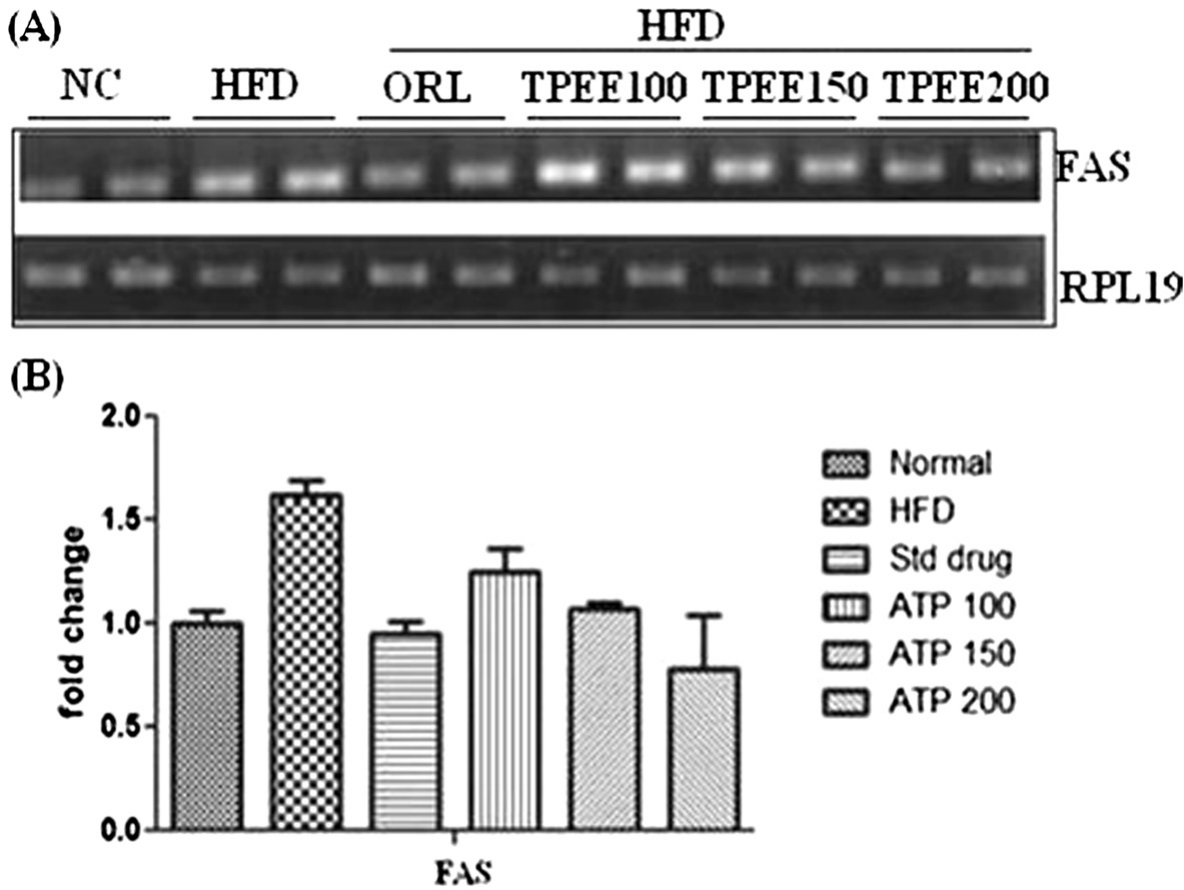
**Effect of TPEE on expression of *FAS *mRNA in control and HFD-fed rats. (A)** Gene expression of *FAS*. **(B)** Densitometric values of *FAS*. The values are expressed as mean ± S.D.

**Figure 3:**
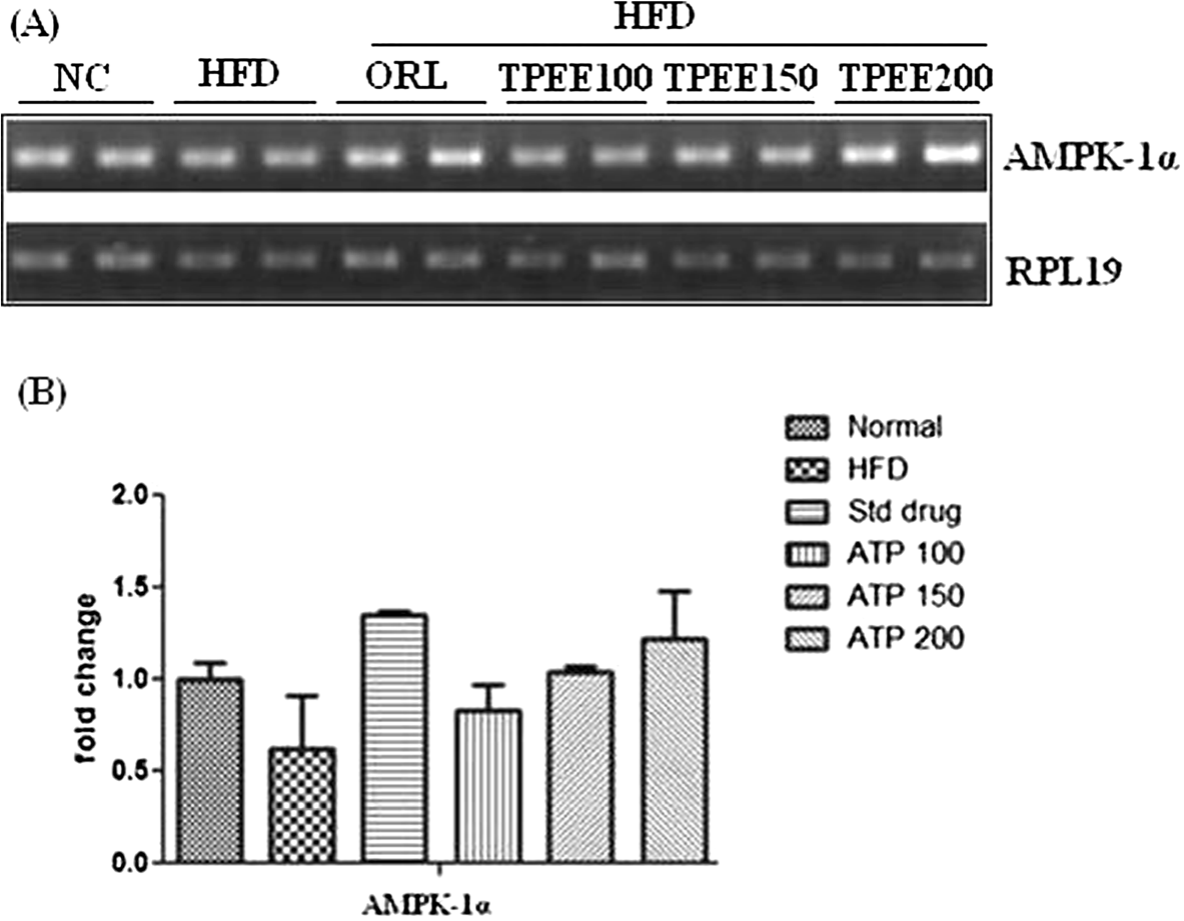
**Effect of TPEE on expression of *AMPK-1α *mRNA in control and HFD-fed rats. (A)** Gene expression of *AMPK-1α*. **(B)** Densitometric values of *AMPK-1α*. The values are expressed as mean ± S.D.

As showed in Figure 2, HFD caused up regulation of *fatty acid synthase (FAS)* expression at mRNA level which was subsequently down regulated by TPEE (100, 150 and 200 mg/kg b.wt) administration (Figures 2A and B). On the contrary, mRNA expression of *AMPK-1α* was down regulated by HFD, but, this was up regulated by TPEE administration in a dose dependent manner (Figures 3A and B).

### Western blot analysis for *FAS* expression

**Figure 4:**
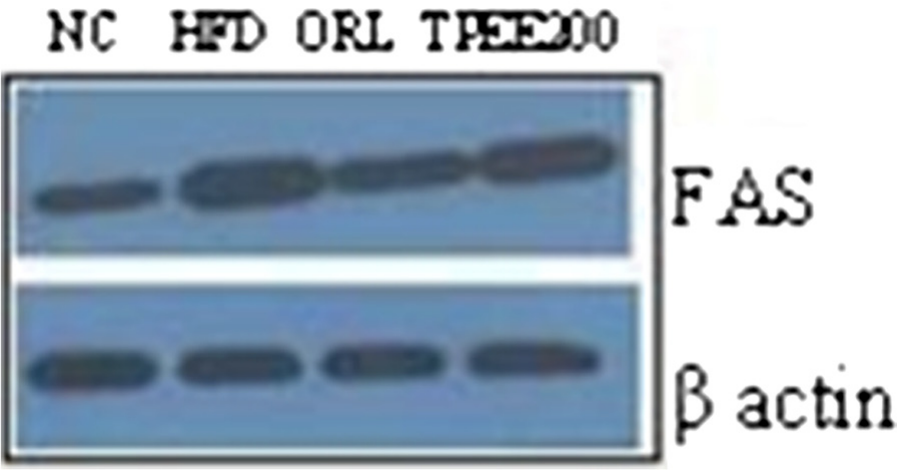
**Effect of TPEE on western blot analyses of *FAS *** **in control and HFD-fed rats.**

To further validate our results, we conducted western blot analysis of *FAS*, a central enzyme in fatty acid synthesis. As depicted in Figure 4, *FAS* expression was up regulated in HFD-fed rats where as HFD + TPEE (200 mg/kg body weight) administrated group showed down regulated level of *FAS*.

### Histopathological studies of liver

**Figure 5:**
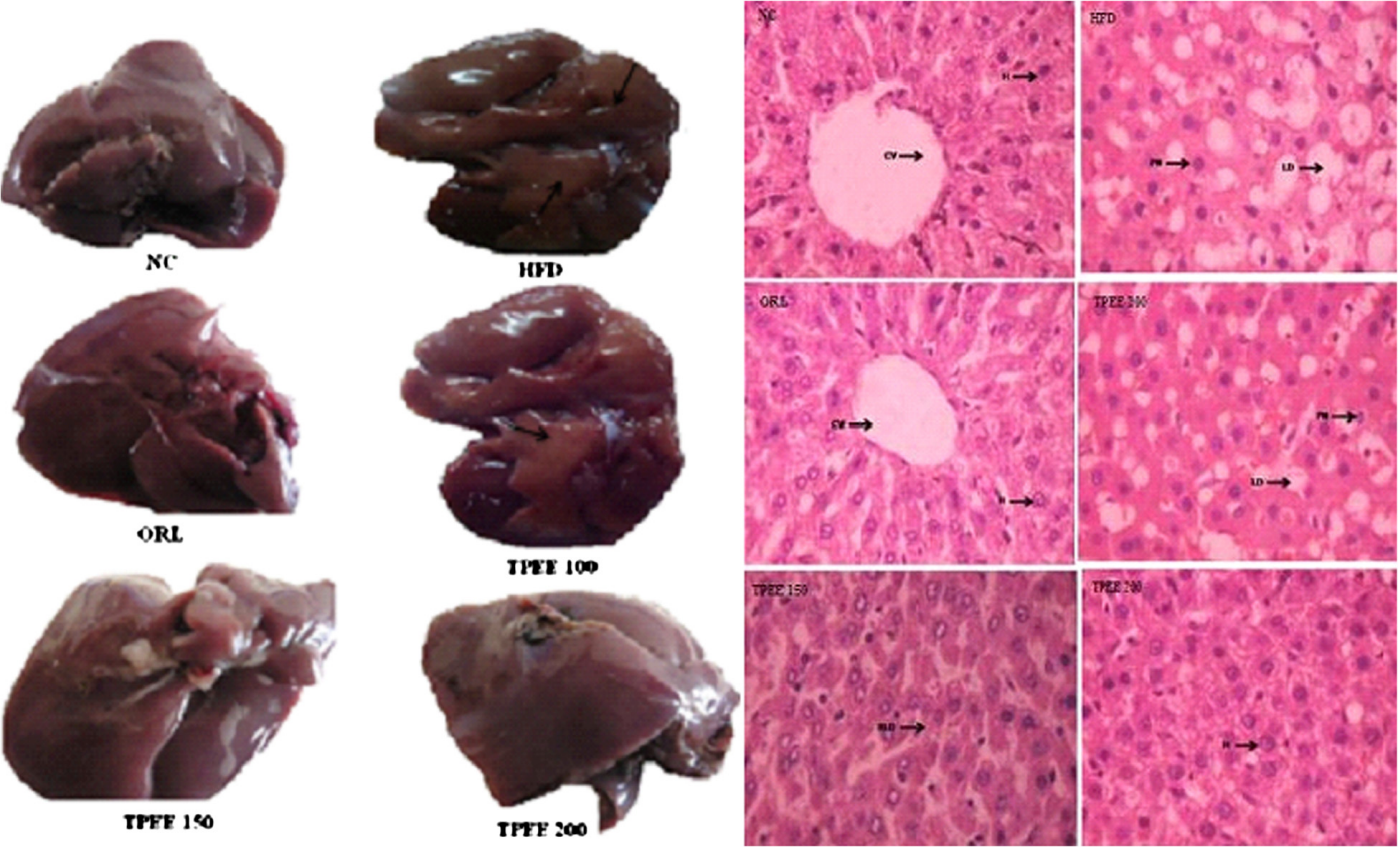
**Effect of TPEE on liver morphology and histopathology in control and HFD-fed rats.** NC: Normal control group, HFD: high-fat diet group, ORL: Orlistat, TPEE: *T. paniculata* ethanolic extract (100,150 and 200 mg/kg b.wt/day).

To evaluate the active role of TPEE on hepatic steatosis we have examined the Hematoxylan and Eosin stained liver microtome sections. HFD-fed control group rats showed higher accumulation of lipid droplets, loss of nucleus, inflammatory cells and severe swelling of hepatocytes, indicating hepatic steatosis. However, HFD + TPEE treated groups showed decreased lipid accumulation, lesser damage and near normal hepatocytes (Figure 5).

## Discussion

The non alcoholic fatty liver (NAFL) and its progressive form non alcoholic steatohepatities (NASH) is a common liver disorder found in dislipidemic and obese conditions. In one end the NAFL, NASH pathological spectrum of steatosis viewed as without inflammation or necrosis, but on the other hand its progression appeared with inflammation, necrosis and liver cirrhosis [16]. In the present study, significant increase in the weights of liver and kidney, elevated hepatic total cholesterol, triglycerides and free fatty acids were noticed in addition to body weight gain in HFD-fed rats. This is in agreement with previous studies on HFD fed rats [5,17]. In the present study we used three concentrations of TPEE (100, 150 and 200 mg/kg b.-wt) on HFD-fed rats. We observed dose dependent activity of these extracts. Among the three doses administered 200 mg/kg b.wt of TPEE was able to significantly (P < 0.05) reduce gain in body weight, organ weights, as well as TC, TG and FFA. This suggests a potential role for TPEE in regulation of fatty acid synthesis, fat accumulation and adipogenesis by modulating key lipogenic factors such as *FAS*, *AMPK-1α* and other transcriptional regulators [18]. Previous reports showed that HFD induces oxidative stress, consequential damage of membrane lipids, proteins and other macro molecules [18]. It is a known fact that in enzymatic reactions, SOD converts superoxide anions to H_2_O_2_ (hydrogen peroxide) and the released H_2_O_2_ can be degraded by CAT to H_2_O [19]. In our study HFD-induced drop in the activity of SOD and CAT but elevated MDA content was noted in HFD-fed rats. But in HFD + TPEE (100, 150 and 200 mg/kg b.wt) administered rats, raise in SOD and CAT activity but fall in MDA level was noticed suggesting potent antioxidant property of TPEE. Elevated SOD and CAT levels in TPEE treated groups could effectively scavenge the reactive oxygen species (ROS) and thus minimized lipid peroxidation. In addition, HFD, devoid of TPEE could cause dysregulated secretion and release of adipocytokines that might contribute to steatohepatites and hepatic inflammation [20,21]. Transcription factors such as *PPARs* and *SREBPs* are involved in regulation of lipid homeostasis by activating the expression of genes required for the synthesis and uptake of cholesterol, fatty acids and triglycerides. Particularly, *SREBP-1c*, one of the pro adipogenic transcription factors, regulates the expression of *FAS* and *AMPK-1α* which play important role in regulation of lipid and carbohydrate metabolism through lipogenesis and uptake of glucose [22,23]. To support our results, we have carried out semiquantitatve reverse transcriptase PCR analysis of important lipogenic genes such as *FAS* and *AMPK-1α* in hepatic tissue. We have found down regulation of *FAS* expression in HFD + TPEE treated groups whose expression was otherwise increased in HFD-fed group. On the contrary, the expression of *AMPK-1α* was up regulated in HFD + TPEE treated groups which was otherwise down regulated in HFD-fed rats. To further confirm the role of *FAS* in lipogenesis and fatty liver development, we performed western blot analysis for *FAS* expression. TPEE (200 mg/kg b.wt) administration has significantly down regulated the expression of *FAS* at protein level in hepatic tissue. This confirms that *FAS* expression was controlled possibly at transcriptional level through *PPARs* and *SREBPs* mediated signaling path way. In fact, our results on TPEE-mediated down regulation of expression of *PPAR-γ* and *SREBP-1c* also supported this event (data not shown here). Furthermore, histopathological examination of liver sections have clearly demonstrated the appearance of normal hepatocytes with reduced lipid droplets in HFD + TPEE treated groups. Further studies are needed to isolate the actual lead molecules from TPEE and to understand the exact mechanism behind their activity.

## Conclusions

Based on our biochemical, histopathological and expression studies, we demonstrate that TPEE potentially attenuates development of non alcoholic fatty liver and obesity.

## Materials and methods

### Collection and extraction of plant material

The bark of *Terminalia paniculata was* collected from Seshachalam forest spread around Tirupati, Andhra Pradesh, India. Its identity was authenticated by a Taxonomist, Department of Botany, at S.V. University, Tirupati, voucher number 136, and a specimen has been preserved at the departmental herbarium. The bark of *T. paniculata* was dried under shade, pulverized to coarse powder and extracted with ethanol. The filtrate obtained was evaporated to dryness at 50-65°C in a rotary vacuum evaporator to obtain a dark colored molten mass.

### Animals

For this study, male Sprague–Dawley rats (*n* = 36), weighing 150-160 g were obtained from National Institute of Nutrition, Hyderabad, India. All rats were housed under 22 ± 2°C temperature, humidity (40-50%) and light–dark cycle (12-12 ± 1 h) and allowed food and water *ad libetum*, for ten weeks. Experimental protocols were followed as per Institutional animal ethics committee guidelines (IAEC) (Regd No: 438/01a/CPCSEA, Date: 17-07-2001). The Committee granted permission to authors and passed resolution to carryout animal work (Resolution No: 36/2012-2013/ (i)/a/CPCSEA/IAEC/SVU/MB-MRG).

### Composition of high fat diet

High fat diet (HFD) was obtained from National centre for laboratory animal sciences (NCLAS), National Institute of Nutrition (NIN), Hyderabad, India. Diet composition is as follows, Casein (342 g), Cystine (30 g), Starch (172 g), Sucrose (50 g), Cellulose (50 g), G.N.Oil (25 g), Thallow (190 g), Mineral mixture (35 g), Vit. Mixture (10 g).

### Experimental design

Rats were randomly divided in to six groups of six each. Group 1: Normal control group (NC), Group 2: High fat diet group (HFD), Group 3: HFD + Orlistat (30 mg/kg b.wt), Group 4: HFD + TPEE (100 mg/kg b.wt), Group 5: HFD + TPEE (150 mg/kg b.wt) and Group 6: HFD + TPEE (200 mg/kg b.wt).

### Measurement of body and organ weights

At the end of the experimental period, rats were weighted and their weights noted. Later, they were fasted overnight, anaesthetized, sacrificed, organs (liver, spleen, kidney and testis were) were surgically removed, wet weights were measured with experimental electrical balance (Shimadzu) and stored at -80°C for further studies.

### Estimation of liver lipid profiles

Liver tissues were washed in ice-cold saline (0.9%), blotted on absorbent paper and weighed. Hepatic lipids were extracted from 1 g liver with chloroform and methanol (2:1 v/v) according to the procedure of Folch [24]. Hepatic cholesterol, triglycerides and free fatty acids were estimated by commercially available kits (Randox Laboratories).

### Assay of Liver antioxidant enzymes and lipid peroxidation

Superoxide dismutase (SOD) activity was determined spectrophotometrically according to a modified method of Beyer and Fridovich [25]. Liver tissue (100 mg) was lysed in isotonic buffer (10 mM Tric-HCl (pH 7.4), containing 200 mM mannitol, 50 mM sucrose and 1 mM EDTA) and centrifuged at 8000 rpm for 10 min. The supernatant was added to a reaction mixture containg PBS (pH 7.8), 0.1 mM EDTA, 12 mM L-methionine, 75 μM nitroblue tetrazolium (NBT) and 2 μM riboflavin to a total volume of 3 ml. The reaction mixture was kept under a fluorescent light for 15 min at 20-25°C and then measured spectrophotometrically at 240 nm. One unit of SOD represents the amount of enzyme required to inhibit the rate of NBT oxidation by 50%. The activity was expressed as units/mg protein.

Catalase (CAT) activity was measured by a modified method of Aebi [26]. Hydrogen peroxide (H_2_O_2_) decomposition by CAT was measured spectrophotometrically at 240 nm. The molar extinction coefficient of 0.043 mM^-1^ cm^-1^ was used to determine CAT activity. One unit of enzyme activity is equal to the micromole of H_2_O_2_ degraded per minute per milligram of protein (min^-1^ mg^-1^).

Lipid peroxidation was estimated by the modified method of Buege and Aust [27]. Briefly the MDA levels were estimated by measuring thiobarbituric acid reactive substances (TBARS) and expressed in terms of malodialdehyde (MDA) content. Before the assay, liver tissues were washed in 0.9% ice-cold saline, blotted on absorbent paper and weighed. Each sample was minced in a Tri-HCl buffer (pH 7.4) and homogenized. After centrifugation at 3000 g for 10 min at 4°C, the clear homogenate was used for biochemical assay. 125 μl of supernatant (S1) was mixed with 50 μl of PBS (pH7.4), 125 μl of 20% trichloroacetic acid containing 1% butylhydoxytoluene and centrifuged (1000 g, 10 min,4°C). Then 200 μl of supernatant (S2) was mixed with 40 μl of HCl (0.6 M) and 160 μl of Tris-Thiobarbituric acid (120 mM) and the mixture was heated at 80-85°C for 10-12 min. The absorbance was measured spectrophotometrically at 530 nm. The MDA levels were expressed as μmol/L/mg protein/mg tissue.

### RNA extraction and semiquantitative RT-PCR

Total RNA was isolated from the liver tissue by using tri-reagent (Sigma-Aldrich, USA) according to manufacturer’s protocol and reverse transcribed to obtain cDNA using DNA synthesis kit (Applied Biosystems, Foster City, USA). 20 ng of cDNA was used for semi-quantitative PCR. The PCR amplification was performed for 38 cycles using the following cycling conditions: 30 sec of denaturation at 94°C, 30 sec of annealing at 59°C and 1 min of extension at 72°C, with following primers: *FAS* (F:ATGTGGTACGGAAGGTGGAG; R: TGGCTACCTTCGTCTGTGTG), *AMPK-1α* (F: GGTCCTGGTGGTTTCTGTTG; R: ATGATGTCAGATGGTGAATT) and *RPL-19* (F: CGTCCTCCGCTGTGGTAAA; R: AGTACCCTTCCTCTTCCCTAT).

### Western blot analysis

Liver tissue protein was extracted with lysis buffer (Sigma Aldrich, USA) and quatified using Bradford method. Equal amount of proteins were resolved on 10% SDS-PAGE gel and transferred to nitrocellulose membrane. To block nonspecific binding sites, blots were incubated at 4°C with 5% (v/v) skimmed milk for 1 hr followed by overnight incubation in primary antibodies of rabbit anti-*FAS* and mouse anti- β actin (Santa Cruz Biotechnology, USA) at 1:1000 dilution. The immunoreactive antigen was then recognized by incubation with Horse radish peroxidase-conjugated secondary antibody (Santa Cruz Biotechnology). Immunoreactive bands were visualized with chemiluminescence detection system (Thermo Fisher Scientific, USA).

### Liver morphology and histopathology

Liver tissues from all groups of rats were collected and kept in 10% formalin solution. A small piece of tissue was sectioned with microtome, fixed on slides and stained using haematoxilin and eosin (H&E) staining procedures and observed under optical microscope.

### Statistical analysis

Results are expressed as mean ± S.D (standard deviation). The statistical analysis of results was done by using *t*-test and one- way analysis (ANOVA) followed by Ducan’s. Values with *p* < 0.05 were considered to be statistically significant.

## Competing interests

The authors declare that they have no competing interests.

## Authors’ contribution

Conceived and designed the experiments: RGM, BM. Performed experiments: RGM, KBS, SD. Analyzed data: RGM, KBS, SD and BM. Contributed reagents/materials/analysis tools: BM. Wrote the paper: RGM, KBS, SD and BM. All authors read and approved the final manuscript.
